# Psychiatric comorbidity in a patient with opsoclonus-myoclonus syndrome. differences in the transition from childhood to adulthood: A case report

**DOI:** 10.1192/j.eurpsy.2021.1729

**Published:** 2021-08-13

**Authors:** F. Casanovas, L. Martínez, R. Cirici, F. Dinamarca, D. García, A. Pérez, L. Diaz, M.T. Nascimento, J.I. Castro

**Affiliations:** Institut De Neuropsiquiatria I Addiccions (inad), Hospital del Mar, Barcelona, Spain

**Keywords:** neurology, Affective disorders, ADHD

## Abstract

**Introduction:**

Opsoclonus-Myoclonus syndrome (OMS), also known as Kinsbourne syndrome, is a paraneoplasic pediatric condition characterized by erratic eye movements and generalized myoclonus. Previous studies have described a wide range of psychiatric comorbidities in children with this syndrome. Cognitive impairment (especially intellectual capacity and language), affective symptoms (irritability, poor mood regulation) and behavioral problems are the most frequent presentations (1). However, there is a lack of literature describing the progression of this symptoms when the patient reaches the adulthood.

**Objectives:**

To illustrate the psychiatric comorbidity of an adult patient with Opsoclonus-Myoclonus syndrome.

**Methods:**

We present one case-report and literature research of the topic.

**Results:**

We present a 18 year old girl diagnosed with OMS and Graves-Basedow hyperthyroidism. During her childhood she started presenting attention and comprehension difficulties. She was diagnosed with an Attention Deficit Hyperactivity Disorder (ADHD) and started treatment with methylphenidate. She completed elementary and secondary education. During the adulthood, the main psychiatric comorbidity was related to affective symptoms. We observed an impaired mood regulation, hypothymia, anhedonia, and frequent episodes of irritability, which persisted after the thyroid regulation. This caused incremented anxious symptoms and insomnia that were treated with mirtazapine and lormetazepam. After some weeks, she fulfilled criteria of a depressive episode and we started antidepressant treatment with vortioxetine.

**Conclusions:**

- Adult patients diagnosed with OMS during childhood can persist presenting ADHD as a comorbidity. - Affective symptoms, and even a major depressive episode, should be considered during the follow-up of this population. Insight of the cognitive limitations could be a risk factor for a depression.

**Disclosure:**

No significant relationships.

